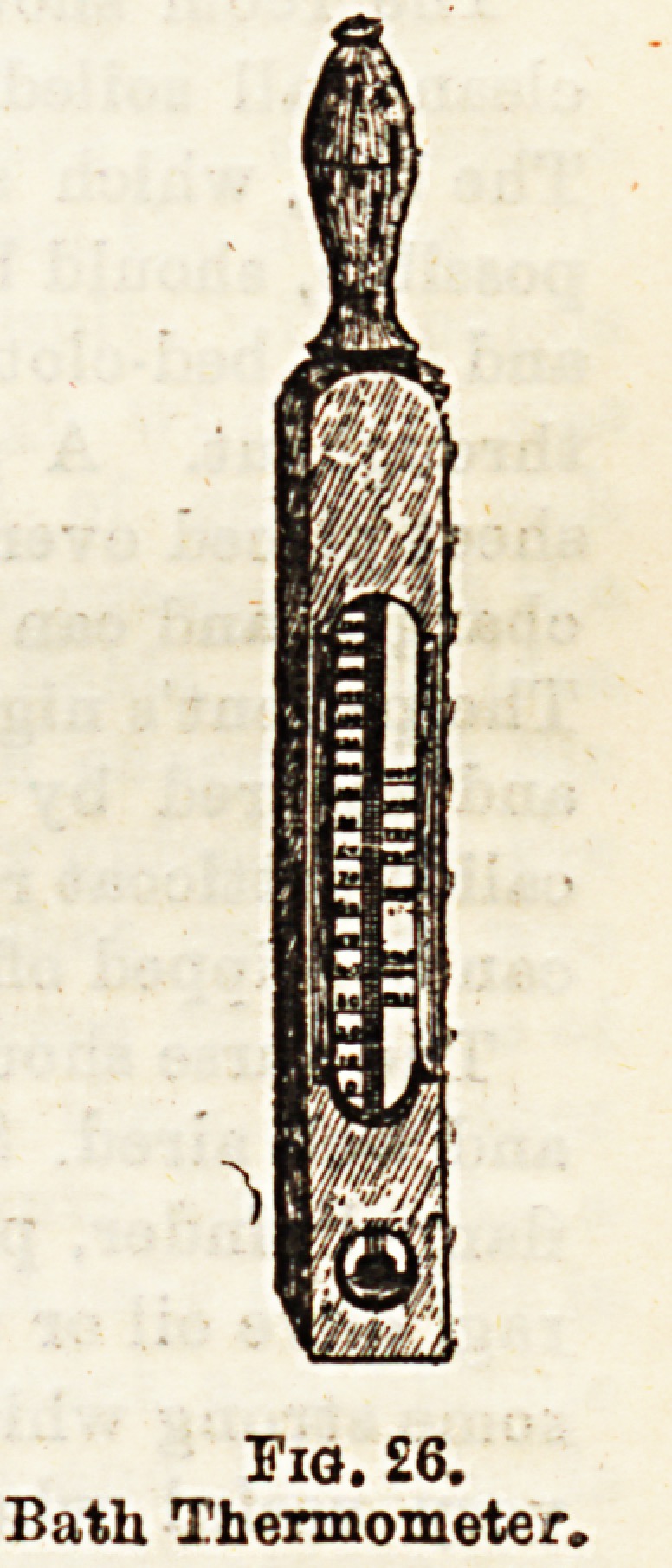# "The Hospital" Nursing Mirror

**Published:** 1896-06-27

**Authors:** 


### "The Hospital" Nursing Mirror.

Being the Extra Nursing Supplement of "The Hospital" Newspaper.

[Contributions for this Supplement should be addressed to the Editor, The Hospital, 428, Strand, London, W.C., and should have the word "Nursing" plainly written in left-hand top corner of the envelope.]

### News from the Nursing World.

### THE PRINCESS OF WALES AT ST. MARY'S HOSPITAL.

The Princess of Wales, accompanied by the Princesses Victoria and Maud, carried out her intention of visiting the wards of St. Mary's Hospital on Tuesday. The Princess stayed for an hour and a half, spending some time in the children's ward, where her visit was a source of much excitement and pleasure to the small patients. Lady Broadbent, Miss Medill, the matron, and Mr. Thomas Ryan, the secretary of the hospital, were presented to Her Royal Highness.

### ON HOSPITAL SUNDAY.

The Rev. C. H. Grundy, preaching at St. Peter's, Brockley, on Hospital Sunday, drew attention to the picture "A Corner in the Children's Ward" in The Hospital Sunday Supplement. "That picture," he said, "reminds us of the loving usefulness and tender work of a nurse's career. What nobler mission could a healthy, refined, intelligent, Christian young woman ask for, than to give her skilled life to the services of the sick, the suffering, and the sad? Only do not let the sarcasm which appeared some years since in one of the papers apply to you: 'Father is getting very blind, and mother is deaf, and the house is so dull that I really think I shall go out as a nurse!' Put home duties first." Mr. Grundy asked his congregation to give liberally, not only for the sake of the poor, but as a "recognition of our obligation here in Brockley to the hospitals which give a man years of training, study, and practice, and send him to give us of his best in skill and attention." The money collected during the day amounted to £120, being £6 in excess of the sum handed over from this church to the fund last year.

### A USEFUL INSTITUTION.

There is on Banstead Downs, near Epsom, an unpretending little institution which does a needful and beneficial work. Six years ago, in a small wooden cottage lent for the purpose, a "Home" for boys was established for the reception of surgical cases, not yet in the convalescent stage, for which fresh air and skilled nursing might be expected to work wonders. A year ago enough money was got together to take the present house, "The Nest," containing twenty beds, for boys under sixteen. It possesses a garden, which on all fine days is full of little invalids sunning themselves on mattresses on the grass, and pony carts are lent by neighbours, and spinal carriages provided to take them further afield. Boys under thirteen are received for a payment of 5s. 6d. a week, those over that age for 7s. 6d. The home is worked voluntarily by a matron and two probationers. The results have proved most satisfactory; wounds, obstinate in hospital wards, have quickly healed in the pure air of the Downs, and now the committee are exceedingly anxious to place the institution on a firmer basis by the purchase of the house and garden, which have been offered to them for the sum of £1,600, towards which they have collected £150. Reports may be obtained from Mr. Leonard Williams, Bank of England, E.C., and visitors will be welcomed at "The Nest" on any afternoon in the week except Wednesdays and Fridays.

### FETE AT MIDDLESEX HOSPITAL.

Great preparations are being made at Middlesex Hospital for the fête on July 1st, in celebration of the opening of the new Convalescent Home at Clacton-on-Sea. Many distinguished patrons and patronesses have promised to support the Duke and Duchess of York on this eventful occasion, amongst others, the Duke of Cambridge, Prince and Princess Christian, the Duchess of Fife, and the Duchess of Teck. The hospital garden, with its fine old trees, will be made very attractive, and will be illuminated in the evening. There is to be an exhibition of pictures in the museum, and a model ward will be on view under the charge of a sister. A concert is also being arranged by Mr. William Ganz, for which Miss Esther Palliser, Madame Alice Gomez, and others have promised their services. We hope the fête will be well attended, and will result in raising a handsome sum for the benefit of the new home. Tickets may be obtained from Mr. F. Clare Melhado, at the hospital.

### ST. PANCRAS INFIRMARY.

A number of visitors accepted the invitation to a general inspection of the St. Pancras Infirmary issued by the Visiting Committee for Friday, June 10th. Tea and coffee were served in the board-room. The wards looked cheerful as always, and were bright with flowers. The day-rooms, partitioned off at the end of each ward, with large windows, are a boon for the convalescents, though it might be wished that something more comfortable than hard Windsor chairs could be provided for their occupants. The nurses' quarters at the infirmary are very good; the large sitting-room on the ground floor is especially worthy of admiration, indeed, it is probably the prettiest and most comfortable room of its kind in London. It costs no more to furnish a room artistically with really comfortable sofas and chairs, covered with art serges of harmonious hues, than to have these same articles of the impossible horsehair variety. This cosy room has four fire-places, and in winter folding-doors divide it into two parts. It has polished floors strewn with coloured rugs, little sociable tables scattered about, and a piano, and with flowers arranged in tall bamboo stands. The general effect is charming. The infirmary committee and Miss Moir are much to be commended on their wisdom in providing this pleasant apartment for the use of their nursing staff.

### DONATIONS FOR THE CHILDREN'S WARD.

Mr. C. F. Cory-Wright, the new deputy-chairman of the Great Northern Central Hospital, has given £1,050 for the endowment of a cot in the Victoria Mary

Ward. This makes the fourth endowed bed for this ward. Mr. Wright has also promised an additional £150 towards further endowment if eighteen other donors can be found to give £50 apiece. At the concert at the Queen's Hall in aid of the Ladies' Association Endowment Fund, we understand that a sum of over £1,800 was presented to the Duchess of York in purses, independently of the proceeds of the entertainment.

### GIFTS FOR THE CHILDREN.

The Paddington Green Children's Hospital has received a donation of £100 from Mr. George Hanbury, who has also promised £100 towards erecting the porch. Mr. Henry J. Ford has promised the terra-cotta material, valued at £50, for the porch.

### ASSISTANT MATRONS FOR METROPOLITAN ASYLUMS BOARD HOSPITALS.

An important and progressive step has been taken by the General Purposes Committee of the Metropolitan Asylums Board. They have issued a recommendation to the effect that when the matron of any hospital under the control of the Board requires more administrative help than can be rendered by a housekeeper, the committee be authorised to appoint an assistant matron, who shall be a trained nurse, at a salary of £50 per annum, with board, lodging, and washing.

### A BRAVE FRENCHWOMAN.

The story of a plucky rescue comes from the Salpêtrière Hospital in Paris. One of the patients, a woman, under the influence of an attack of delirium, climbed over a window-ledge of the ward (which was on the top storey of the building), and succeeded in reaching the roof. An *infirmière*, Mme. Petit-Barrat, rushed in pursuit, and cleverly contrived to bring the poor woman back in safety. This brave nurse has received many compliments on her courageous act.

### A TRIBUTE TO LADY DUFFERIN.

A handsome clock and candelabra have been presented to Lady Dufferin on her departure from Paris, by English and American lady residents. It is also proposed, by way of an additional tribute, to subscribe a sum of money to be devoted to the Victoria Home and another charitable institution in Paris, founded by Lady Dufferin, and in which she takes the deepest interest.

### NEWS FROM GLOUCESTER.

There has been a large drop in the number of cases of small-pox notified in the last week or two. The second hospital at Hempstead has now been closed, and the good sisters from Clewer who were working there have transferred their services to the Isolation Hospital in the Stroud Road. Here matters have been reduced to order under the rule of Dr. Brooke and Miss Walker, and now the wards are no longer crowded. A good deal of interest has been taken in the prosecution by the sanitary authority of Mr. Spring, pastor of the East End Tabernacle, on a charge of going about while still infectious. Mr. Spring had a mild attack of the disease and was treated by a gentleman from Leicester, who has undertaken to cure small-pox sufferers in a few days by his "hydropathic" treatment, and whose ministrations have—been consequently popular and dangerous during the outbreak. A fine of £5 and costs was ultimately imposed, and a second charge withdrawn on payment of costs.

### GUY'S HOSPITAL AND THE ROMAN CATHOLIC LEAGUE.

A paragraph has been going the round of the papers to the effect that the executive of the Roman Catholic League have protested against the rules which "forbid the employment of (Roman) Catholic nurses at Guy's Hospital." As a matter of fact, there are no such rules. At Guy's Hospital, as at King's College Hospital, and at others, as a matter of discipline the nurses are expected to be present at prayers, and as Roman Catholics are forbidden to be present at any religious ceremonial save that of their own Church, they do not choose these hospitals for training. We hold it to be a pity that there should ever be compulsory attendance at religious services, which should be, beyond all acts of human life, spontaneous; but it should be clearly understood that Roman Catholics are not "forbidden" from participating in the advantages of training at Guy's except from this cause. One may be pardoned a feeling of deep regret that "denominationalism" should thus prevent fellow-Christians from worshipping in concert.

### SHORT ITEMS.

A District Nursing Association has been started at Blackburn.—The president of the Queen's Jubilee Nursing Institute at Southampton has issued a special appeal for funds to help the committee to carry on the district nursing work. Subscriptions will be thankfully received by Dr. Gwillim, hon. secretary, 3, Laura Place.—At the close of the Nursing Exhibition, lately held at St. Martin's Town Hall, Mrs. Bedford Fenwick was presented with an album containing the press notices which had appeared of the exhibition on behalf of a number of nurses and others interested in the enterprise.—The Borough of Newport (Isle of Wight) Nursing Society has ceased to exist. Miss Fullagar, who has worked indefatigably for the last five years as nurse, has been obliged to resign on account of failing health.—At recent medical examinations in Japan twenty-seven Japanese ladies presented themselves amongst the candidates for qualification to practise. This is a very progressive movement, and one upon which the women of Japan must be warmly congratulated.—The annual meeting of the Working Ladies' Guild (351, Brompton Road) was held on Friday, June 19th, at 1, Upper Belgrave Street, by permission of Lady Susan Leslie Melville. The Rev. and Hon. E. Carr Glyn, vicar of Kensington, presided, and the Bishop of Southwark gave an address on the work of the guild.—The annual meeting of the Society for Promoting the Employment of Women was held at the office, 22, Berners Street, on Wednesday, June 10th. Lord Stanmore presided.—We are requested to state that no definite arrangements have yet been made with reference to the reception of the Queen's Nurses by Her Majesty at Windsor, on her return from Balmoral. Due notice will be given as soon as details and date are fixed.—The concert at Stafford House on behalf of the Free Home for the Dying at Clapham has realised the sum of £237 5s. 6d., in addition to which a lady has sent a cheque for £500 to the honorary treasurer, Mr. W. Hoare.

### Hygiene: For Nurses.

By John Glaister, M.D., F.F.P.S.G., D.P.H.Camb., Professor of Forensic Medicine and Public Health, St. Mungo's College, Glasgow, &c.

### XII.—SICK-ROOM TEMPERATURES—REGULATION OF HEAT OF SICK-ROOMS—BATH TEMPERATURES.

The temperature-scale of the clinical thermometer usually ranges from 92° to 112° Fahr. The extreme points are obtained by placing the instrument in liquids, in which a standard instrument records these temperatures. It is then graduated between these points in the manner already indicated. It must not be assumed that every instrument which may be bought is accurate. Most of them, indeed, have errors of calibration, or graduation, or both. It is important to have an accurate instrument, although an instrument which is not precisely accurate will register variations of temperature in a relatively accurate manner. If, however, its initial accuracy be at fault, any given single temperature will either be too high, or too low. To secure an accurate thermometer, it ought to be corrected against standard instruments, as is done at Kew Observatory. The modern instrument is self-registering; that is to say, when the mercury has attained the highest point of the reading, the index column remains in this position, while the rest of the mercurial column recedes into the bulb. This is accomplished by intervening a minute bubble of air between the main and the index column. Before another reading can be taken, the index column must be shaken below the level of the normal temperature reading, this being done by holding the non-bulbous end of the instrument in the hand, and giving it one or more shakes in the air. This design of instrument enables the observer to remove it from the body of the patient before the reading is taken; whereas, with the non-registering form, the reading has to be taken with the instrument in position. Quick temperature-registration instruments are made by having the bulb thinned and elongated so as to present a larger mercurial surface to the heat-giving source. Spirally-coiled instruments, for taking mouth-temperatures, are sometimes used, but they have no merit over the elongated form.

The normal temperature of the human body in health, taken in the armpit, is 98·6° Fahr., or 37° Cent., or 29·6° Reau.; taken in the rectum or mouth it is 99·2° Fahr., or 37·3° Cent., or 29·9° Reau. A temperature registered above these points is called a hypernormal temperature (Greek *hyper*=above); and one below, a subnormal temperature (Latin, *sub*=below).

| | |
|---|---|
| Temperature of collapse | =95° to 97° Fahr. |
| Subnormal temperature | =97·6° Fahr. |
| Normal temperature | =98·6° Fahr. |
| Subfebrile or slightly hypernormal temperature | =99 to 101° Fahr. |
| Febrile temperature | =101·5 to 103° Fahr. |
| Highly febrile temperature | =103·5° to 104·5° Fahr. |
| Hyperpyrexial temperature | =105° to 106·5° Fahr. and upwards. |

The temperature of the sick room ought entirely to be regulated by the comfort of the patient. A routine temperature which fulfils this condition for convalescents, elderly persons, and young children is one from 60-65° Fahr. Patients who suffer from fever generally, or from one of the specific fevers particularly, experience more comfort when the room is about 55° Fahr. than when it is higher. In addition to temperature, comfort is materially added to certain patients if the humidity of the air receive special attention. This is true of pulmonary diseases generally, but especially of "throat" and "croupy" cases. It is practically impossible to reduce the amount of watery vapour in any atmosphere, but it is possible, and often advantageous, to increase it. This is the reason for the croup kettle and other steam-generating apparatus for the sick-room. The thermometer, therefore, ought to have a prominent place in every sick-room and to receive the careful periodic attention of the nurse. (Fig. 24.)

Fig 25 is the wet and dry bulb thermometer, by means of which, on reference to a table of instructions, the amount of moisture in the atmosphere may be determined, in addition to the temperature. The little vessel at the bottom of the thermometer on the right contains water, which, by capillary action, passes up the strands of wick from the vessel to the bulb of the instrument, and so keeps it wet; hence the name wet bulb. The other thermometer has no such arrangement, hence the name dry bulb. The temperature of the sick-room requires careful attention, especially about midnight and the early hours after midnight, and four a.m., for at these times the bodily temperature falls lowest.

The regulation of heating of ordinary sick rooms, especially at night, is a somewhat difficult task. To lower the temperature, beyond the ordinary method of opening the window and door, as already described, there is little to suggest. Careful attention to firing, especially when lights are burning, will make the task easier. Direct heat from the fire may be prevented reaching the patient by the simple interposition of a screen.

Fig. 24. Thermometer.

Fig. 25. Wet and dry bulb Thermometer.

Fig. 26. Bath Thermometer.

Baths and Bath Temperatures.—Both in hospital and private nursing the bathing and sponging of patients are important items of the nurse's duty. Here, also, the thermometer is all important. Fig. 26 shows a bath thermometer. It is important, alike for the physician who orders the bath, and for the nurse who is to carry out his instructions, to understand clearly what is meant when a particular bath is called for. We speak, for instance, of a cold, a tepid, a warm, and a hot bath. What is meant by these respective terms? They are all relative terms; for example, a patient with a temperature of 104° Fahr. will feel less cold on being put into a bath at 65° Fahr. than will a person whose temperature is normal; and tepid water will feel warm, and warm water hot to a cold body. It is for this reason that the rule-of-thumb method of testing a bath temperature by passing the hand through the water should, once and for all, be discarded as untrustworthy, for the amount of hotness or coldness which is perceived by the testing hand will depend entirely upon the temperature of the hand at the moment of testing. Again, patients have been scalded by having been put into baths by careless attendants who had not used the thermometer. For these reasons, therefore, the bath thermometer should always be used before the immersion of the patient. To return, however, to bath temperatures. While a cold bath may mean anything between the melting point of ice and 65° Fahr., it is necessary when a cold bath is ordered, as is now not infrequently done in certain febrile diseases, that the physician ought to signify the temperature of the water to be used. If this be omitted, the nurse ought to have some knowledge of ordinary bath temperatures and be able to apply it.

The following table may serve as a useful guide in this matter:—

| | | |
|---|---|---|
| Cold Bath ... ... ... | 60—65° | Fahr. |
| Tepid ... ... ... | 86° | " |
| Warm ... ... ... | 95° | " |
| Hot ... ... ... ... | 104° | " |

Routine bath temperature for infants and young children, 85° to 90°.

Bath thermometer always to be used before immersion of patient.

It is always safer for convalescents, elderly people, and persons generally who cannot afford to part with heat freely, to wash in the winter season in tepid or warm water. Much nonsense has been written regarding the innervating effect of cold water and the enervating effect of warm. The former may so act for the vigorous and healthy, but it does not for a large number of persons, particularly those whose heart's action is slow and feeble, or who are below par generally. Sponging of the body at intervals with a damp sponge wrung out of tepid water is very grateful and soothing to the feverish patient, and it will often be the means of inducing sleep, and certainly restfulness in delirium, in feverish patients. As a general principle, the temperature of the water used ought to be regulated by the amount of fever present, viz., the higher the fever the cooler the water. The accompanying comfort will not only be more marked at the time, but will also be more lasting.

### Midwifery Papers.

### IV.—THE LYING-IN-ROOM REQUISITES. EXAMINATION OF PATIENT.

When a midwife is called to a case she should go at once, and after convincing herself that her patient is in labour, which is progressing favourably, she should see that everything she will need for the mother and child during the labour is to hand in the room.

The room should be airy and quiet, and, needless to say, clean; all soiled articles must be immediately removed. The bed, which should be without curtains or hangings if possible, should be supplied with a firm and level mattress, and the bed-clothing should be warm and light and clean throughout. A good-sized mackintosh covered by a draw-sheet placed over the bottom sheet protects it from the discharges, and can be easily removed when the labour is over. The patient's nightdress should be rolled up above her hips and secured by safety-pins, and she should wear a loose calico petticoat reaching from the waist to the knees, which can be slipped off without any difficulty.

The nurse should see that all the baby's clothing is ready and well aired. She will need for it a gown, flannel petticoat, flannel binder, pilches, shirt, flannel head-shawl, soft white rag, olive oil or vaseline, Fuller's earth or starch powder, and some strong white thread or silk. A large flannel apron is very useful when bathing baby. For the mother's use a binder, diapers, absorbent cotton wool, large pins, bed-pan, and feeding cup should be in readiness. The binder most in use in lying-in hospitals is one made of the ordinary huckaback towelling, and is generally one and three-quarters to two yards in length and half a yard wide. It is more comfortably and firmly secured by using large-sized ordinary pins instead of safety. Pads of wood-wool enclosed in a layer of mercurial gauze are also used instead of the ordinary diaper.

The room should be provided with hot and cold water, soft towels, pail, two or three large basins, olive oil, and soap. All these articles should be in readiness; there should be no rushing away in search of them when they are most needed, to the discomfort and perhaps danger of the patient.

The nurse will need in her midwifery-bag a supply of the ergot powder and tincture of carbolic acid, perchloride of mercury, permanganate of potass, boracic, vaseline, iodoform, catheter (soft, No. 8), surgical scissors (blunt-pointed), Higginson's syringe with glass vaginal tubes, clinical thermometer, bath thermometer, needles and white thread or silk, safety and ordinary pins, and absorbent cotton wool.

During the first stage of labour the patient should be allowed to walk about her room, resting at intervals. She should be encouraged to take light nourishment in the shape of milk pudding, beef tea, or a cup of tea or coffee. A warm loose dressing-gown should be worn over the night-dress and petticoat. The nurse should chat with her patient, carefully avoiding any emotional or exciting topic. Of course, no nurse who is worthy the name discusses with a woman in labour the difficult cases she has attended before, and the dangerous complications through which she has dragged previous patients by her unrivalled management and skill! Such nurses, and I sincerely hope there are not many, do not inspire confidence in themselves or their nursing. In the lying-in room the nurse should always wear a clean washing-dress, the sleeves being open or sufficiently loose at the wrist to be pushed well out of the way up the arm. A large white apron protects the dress, and can be easily changed when soiled. In hospital we are taught to keep our nails short and well trimmed, and on entering the labour ward we are told to wash our hands and use the nail brush. We then dip them in a solution of perchloride of mercury ($\frac{1}{1000}$) before we are permitted to examine our patient. I have sometimes noticed in private nursing that those most necessary precautions are neglected, even by nurses, who, one would suppose, could appreciate the value of them. One of the first facts to be learnt in midwifery is that a nurse can never be too clean or too careful in her work.

On Examination.—When a midwife is asked some time during the pregnancy to undertake a confinement case she should examine her patient to convince herself—(*a*) that pregnancy exists, (*b*) that there is no condition present to prevent natural labour. She should carefully note the presence or absence of the usual signs of pregnancy, and should make an examination of the abdomen and vagina. To examine the chest and abdomen the patient should be placed on her back, with a pillow supporting her shoulders; the knees must be drawn up to relax the abdominal walls. Especially when listening for the fœtal heart-beats the room should be perfectly quiet, as the sounds are difficult to discover if the abdominal walls are thick or pendulous. When making a vaginal examination the patient should be placed on her left side near the edge of the bed, with the head low, and the knees flexed on the abdomen. The midwife should note the appearance and condition of the vulva and vagina, and if she suspects the existence of a contracted pelvis, or any other obstruction to normal labour, she should request the advice of a medical man. In women who have suffered from spinal curvature or hip-disease, or are ricketty dwarfs, the pelvis is generally contracted or malformed, and the difficulty in delivery will be too great for treatment by a midwife.

An important point to remember in examination is that no force should be used. A rough vaginal examination especially might induce premature labour or injure the soft parts examined.

### Cottage Nurses.

At the recent annual conference of the Affiliated Benefit Nursing Associations held at Stafford House, Miss Broadwood gave an address in which she reviewed the work accomplished by the parent association started 13½ years ago by herself "on a system which many persons of experience had said was unworkable." To-day there were 88 similar associations working in different parts of the Kingdom, employing between them about 240 cottage nurses: twelve of these associations were in Surrey, and twelve in Sussex, besides the parent one, which extended over a wide district in both these counties; in Oxon, ten; in Gloucestershire and Warwickshire, five each; in Hants, Berks, and Yorks, four each; in Somerset, three; in Kent, Wilts, Devon, Worcestershire, Salop, and Herts, two each; in Cornwall, Cheshire, Flintshire, Lancashire, Glamorganshire, Stafford, Bucks, Notts, Rutland, Essex, Suffolk, Durham, Northumberland, Ross, and Sutherland, one each; the adjoining corners of Northampton, Oxon, and Warwick having one between them. She feared she must appear to some persons most disagreeably unyielding on certain points because she refused to be convinced of the advantages of certain changes which had been from time to time pressed upon her by persons not practically acquainted with the work in which she was interested. It was thirteen and a-half years since it had been urged on her by a member of the Matrons' Aid Society that if maternity cases were nursed at all it should be by certificated midwives only. It was seven years since her old friend, Sir Rutherford Alcock (who had most kindly exerted himself to show his sympathy by his presence this day), had placed before her the rules of the Jubilee Institute, then about to be published, suggesting affiliation if possible. But after discussing the *pros* and *cons.* with him and with another old friend and councillor of hers, Sir W. Bowman, she had pointed out that the year's training in a general hospital, which the Jubilee Institute makes a *sine quâ non*, debarred this affiliation. She felt confident then, as she did now, that cottage nurses, as they are, do their special work excellently and satisfactorily, that they must be recognised, and should be inspected. Inspection would be desirable if it could be arranged free of cost to the associations, although she confessed to attaching less importance than some persons to the formal inspection of the nurses by a highly-trained lady nurse, who at most can only see each nurse and look over her reports every six months or so, and who can judge less well of her work in any particular case from her reports, than the member of the committee who had every opportunity of seeing her at work, and the medical man who was in charge of the case. What she aimed at more than inspection was examination. It was just six years since a member of the Jubilee Council assured her that none but lady nurses should be employed, with charwomen to follow, and that the women should not be called cottage nurses but cottage helps. Her answer to that was, "What are those who tend and nourish children, who wait on the sick, commonly called? The cottage nurses were doing much good and no harm, and there was no reason whatever why any slur should be cast upon these good women who had devoted their time to ministering to the suffering." No one could be more jealous and careful than she that the name "cottage nurse" should be known to apply solely to those women who have been registered as qualified to act in that capacity. Whenever they are sent for training in maternity nursing and instruction in the elements of general sick nursing, it is arranged that their certificates shall be withheld until such time as each one has fulfilled her term of engagement (of three or four years), with one or other of the associations to the satisfaction of the medical men, the patients, and committee, and as each certificate is endorsed with a reference to the registry kept at the central office, it is impossible that any cottage nurse so registered should pass herself off successfully as more competent or more skilled than she is. If all the cottage nurses are not registered it is not for lack of facilities. If a central body would examine the nurses on the special instruction which they have received, it would be a great help, and the associations would be enabled to judge still better than at present of the relative worth of individual cottage nurses, and of the various training fields to which they are sent. The value of these latter depends greatly upon the number and variety of cases that can be seen in a given space of time. In lying-in hospitals from 30 to 40 maternity cases can be seen in six weeks; in overcrowded town districts and large infirmaries where there is much sickness as well, both the maternity nursing and the elements of general nursing can be learnt more rapidly than in a country town or in a cottage hospital. Thus it is absolutely necessary to be at liberty to have various periods of training. In some instances twelve to fifteen months in one place would be followed by a six weeks' course in a lying-in hospital, while in others three to six months in a crowded district supplies the needful number of cases and experience. On the previous day the general committee had decided: "That inasmuch as most satisfactory work has been done for benefit nursing associations by nurses who have undergone very different periods of training, it is inadvisable to interfere with the discretion of those arranging for the training, as they have the advantage of the advice of the medical men under whom the nurses will work, and also the experience of former results with pupils in the different training fields." Years ago when she was anxious to find out where the elements of general sick nursing could be taught, Sir W. Bowman suggested that the large workhouse infirmaries would be good fields. This idea was scoffed at by the trained nurses then in charge, but Sir W. Bowman recommended "pegging away quietly" at the general public, and she was glad to feel that opinion was coming round to this view, and that they were on the eve of getting one or two infirmaries opened to them. Another point urged had been the working on county lines. As a Borderer, in more senses than one, she could only say that it would have sorely hampered the very quiet and humble start of the Ockley Association had she and her friends been ambitious to form themselves into anything more than a private body for helping their poorer neighbours. Her father's estate and the poor friends whom she was specially concerned to help being scattered over the verge of Surrey and Sussex in several parishes, it would have been impossible to specially localise the work even if it had seemed desirable to do so, and she was unable to see that cottage nursing is spreading more, or even as much, in those counties which are working on county lines than in others. In a word, she was averse to advocating the changes suggested until those who believed in them had proved them to be changes for the better. Miss Broadwood then read a *résumé* of the report of the second year's work, which stated that 57 nurses had been sent into training and placed on the Cottage Nurses' Registry, five supplied permanently who had not been trained for the special work, and 29 supplied for emergency cases. There were now 66 affiliated associations and 22 non-affiliated, making a total of 88.

### Wants and Workers.

*Holidays.*—Miss C. Kennedy, 2, Natts Cottages, Chase Side, Enfield, would like to communicate with the "Member of the R.N.P.F." who is thinking of going to Portrush for her holidays.

*Work Wanted.*—A trained nurse, who is obliged to give up nursing work on account of ill-health, would be glad of any orders for uniform aprons or hand-knitted vests and undercloth. Write to Nurse E., 61, Harrowby Street, Princes Road, Liverpool.

### Notes from Melbourne.

(COMMUNICATED.)

A LARGE addition has been made to the infirmary department of the Women's Hospital at Melbourne, including a new operating theatre, a large general ward, and small wards for special cases, and the new wing was formally opened on Tuesday, April 28th, by the Governor's wife, Lady Brassey. The new building has cost about £1,600, mainly contributed by the women of Victoria, as the result of a self-denial movement. It is estimated that the new wards will add about £500 a year to the expenditure of the hospital.

For some years past it has been the custom at the Melbourne Hospital to appoint as resident medical officers those six students at the University who in the final examination for the M.B. degree come out first in the honour list. Amongst the six this year were two ladies—Miss Alfreda Hilda Gamble and Miss Janet Stocks Greig—who both desired the positions. It may be explained that no salary is paid to these six juniors; but the appointments are much coveted for the sake of the experience. The committee of the hospital had many anxious consultations on the matter, and even sought the advice of the faculty of medicine of the University; but that body unkindly declined to give advice, though it expressed a fear that the appointment of women resident medical officers in the Melbourne Hospital would impair the efficiency of the clinical teaching. The end of the matter was that the ladies were duly elected by a majority of the committee. They began work in the hospital on Saturday, April 25th, and the result of the experiment is awaited with much interest. A feature in the matter was the pronouncedly antagonistic position taken up by one of Melbourne's two morning daily newspapers, which published inimical paragraphs every day, and on the morning of the election even had an absurd account of the utterances of some of the patients who did not like the prospect of being attended by women doctors. These tactics, which were treated with the silent contempt they deserved, were carried to the length of publishing a long account of the newly appointed ladies' first day's work at the hospital, wherein the most alarming case seems to have been an "alcoholic" who had to be steadied with the electric battery.

The committee of the Children's Hospital wish to found another permanent cot to be called the Victorian Children's Cot, and for that purpose they invite 500 young Victorians, from two to 14 years of age, to collect each one guinea in small sums; the 500 guineas are expected to reach the hospital by June 25th, so as to be in time for mention in the annual report.

The Victorian Eye and Ear Hospital has also been enlarging its accommodation, this being possible out of a donation of £2,000 presented by Mrs. Bowen, from moneys left by her late husband, Dr. Aubrey Bowen, for charitable purposes. Dr. Bowen was more than twenty years in connection with the Eye and Ear Hospital, and the new wing has been named after him. The committee have now built over all the available land, and the hospital can only be further enlarged upwards. The garden, which was a great help and a delight to the child patients, has been lost by the enlargement.

Typhoid is now (May) abating with the increase of the cold weather, but the late season has been the worst for four years past. All the hospitals have been full, not only the Melbourne hospitals but all the country ones as well. Early in March there were 69 cases of typhoid in the Melbourne Hospital alone. In February the Alfred Hospital opened sixteen more beds for typhoid patients. When the deep drainage scheme is accomplished in Melbourne it is confidently anticipated that typhoid will decline; meantime, Dr. Jamieson, the City health officer, has made an interesting comparison of the relative rates of mortality of typhoid between Melbourne, London, and the principal English towns. Melbourne mortality is 3 02 per 10,000, which Dr. Jamieson thinks is not actually high for an undrained town.

The cold bath treatment of typhoid has been pursued for the past nine years in the Brisbane Hospital (Queensland), with the result of a strikingly diminished mortality, and recently an agitation was set on foot to have it introduced into the Melbourne hospitals, where it has not been hitherto practised. The newspapers interviewed a number of local medical men on the subject, but most of them were of opinion that the climate of Melbourne, so much colder than that of Brisbane, rendered the cold bath treatment very risky, and the matter "fizzled out" in a few newspaper letters.

The fourth session of the Intercolonial Medical Congress was held in February in Dunedin (New Zealand), and attended by about one hundred delegates. The next is to be held in Brisbane in the early part of 1897, when Dr. John Thomson, of that city, will be the president.

Quarantine is, and must always be, a matter for serious consideration to Australians, and there is no doubt that rude as are our present appliances, the rigorous method in which quarantining has been carried out, at least in the port of Melbourne, has saved the country from many pests. The consequences that would follow the turning loose of two or three cases of small-pox, for instance, in one of our hot summers are alarming to contemplate. Thus, a conference of representatives of health boards from several colonies that met in Melbourne in March gave a large share of attention to quarantining, and passed a number of resolutions of a stringent character which are likely to bear fruit in the establishment of medical experts and quarantine stations at Albany (west), Adelaide (south), and Thursday Island (north-east), and later at Palmerston, on the north of Australia. Vaccination is insisted upon, and absolute power over ships and passengers would be given to the medical men in charge. The concluding resolution was that another intercolonial conference be held about a year hence to see what has been the practical outcome of the resolutions passed. The various colonies are to bear the expenses of the proposed system of federal quarantine on the basis of their respective populations.

### Maternity Charity and District Nurses' Home.

ON Saturday last the Countess of Winchilsea presided at an "At home" at the Maternity Charity and District Nurses' Home, Howard's Road, Plaistow, E. Amongst the visitors were the Duchess of Sutherland, Lady Selborne, Lady Thorold, the Archdeacon of Essex, and the Earl of Winchilsea. The object of the gathering was to interest people in the work of the charity, and to point out the inconveniences of the nurses living in nine different scattered houses. The visitors inspected the various houses, the many disadvantages of which were very evident. It is hoped to raise a sum of £4,000 for the building of a suitable home, where they may live all together. On the previous Thursday a drawing-room meeting was held at 35, Park Lane, by kind permission of the Countess of Grosvenor and Lady Ebury, which was well attended, amongst the speakers being the Bishop of Colchester, the Earl of Winchilsea, the Rev. T. Darwin-Burton, and Mr. H. Phillips. The meeting was for a similar object. Donations should be sent to the treasurer of the charity, Mr. Robert Williams, or direct to the bankers, Messrs. Williams Deacon and Co., 20, Birchin Lane, E.C.

### Women as Sanitary Inspectors.

We have always been in favour of women being allowed a fair field and no favour in regard to all such positions as they can fill as well as men. Many positions have hitherto been closed to women for which they are even more fitted than men, and among these are various kinds of inspectorships, especially such as have to do with trades in which large numbers of women and young persons are employed. Even as sanitary inspectors there seems a field for women. So long as the management of the home remains in the hands of women — which we may presume will be for a long time yet to come—the duty of inspecting the sanitary details of the home may fitly fall to women also. But there are details in the work of some of the sanitary inspectors who are appointed to carry out the provisions of the bye-laws framed under the Public Health Act, which we can hardly bring ourselves to look upon as proper work for women. At a meeting of the Vestry of St. George the Martyr, held on June 16th, it was decided that a lady sanitary inspector should be appointed. So far so good. But what is this lady inspector to be asked to do? We understand that the appointment was made partly in consequence of the County Council having written urging the carrying out of the necessary inspection under the tenement bye-laws, which relate to houses let in lodgings to more than one family, and that this inspection will involve visiting by night as well as by day some of the worst slums in London, inhabited by thieves, prostitutes, and such-like difficult characters. It certainly may be questioned whether this is the sort of sanitary inspection for which women are as well fitted as men. It must be borne in mind that if the work is to be efficiently performed the sanitary inspector will in many cases have to prosecute both landlords and lodgers, and that as the latter can hardy ever pay fines, they will have at her hands to be sent to prison. Thus she must either neglect the work or must make for herself enemies, into whose hands she must, night after night, deliver herself unarmed and unprotected in the execution of her duty. While human nature remains as it is, and while the dark alleys and unlighted staircases give such opportunity for revenge and spite as they do, it is impossible to hide from one's self the fact that if the work is properly done it involves a certain amount of personal risk, and although we know that there are brave women who are willing to go anywhere we may doubt the propriety of sending them. It would be very wrong to argue from nurses and district visitors to inspectors of lodging-houses. Nurses and district visitors undoubtedly often go to some of the worst parts of London and meet with no harm. But they always go as friends and helpers. The inspector, however, is partly an agent of the law, and partly a spy upon their inner life, and in neither character is he loved. We should be sorry to see the admission of women to posts for which they are well fitted brought into disrepute by their accepting appointments which are obviously unsuitable for them.

### Death in Our Ranks.

We much regret to announce the death of Sister Rose Page, on June 17th, of heart disease. Sister Page received her training at University College Hospital, and for the last seven years has had charge of the women's surgical wards of the East Suffolk Hospital, Ipswich, where she has won for herself the esteem and respect of patients and fellow-workers.

### Everybody's Opinion.

[Correspondence on all subjects is invited, but we cannot in any way be responsible for the opinions expressed by our correspondents. No communications can be entertained if the name and address of the correspondent is not given, or unless one side of the paper only be written on.]

### THE JUNIUS S. MORGAN BENEVOLENT FUND.

"Policy 1,976" writes: Considering the enormous amount of money given yearly to our sick and helpless sisters by the Junius Morgan Benevolent Society, and of the additional number which seek its benefit through no fault of their own, might I suggest to my sister nurses, through The Hospital, that we give something a year to the Junius Morgan Fund, even be it ever so little? Almost all our working members could afford one shilling a year—some could give more—and I am sure we nurses ought to help. I enclose 2s. 6d., and trust many of my fellow-sisters will think it right to help according to their means.

[We have pleasure in publishing the above letter, and learn from the hon. secretary of the Junius S. Morgan Benevolent Fund that she will be glad to receive contributions from any nurse desiring to follow the suggestion of Policy 1,976. We are desired also to remind nurses that the Fund was originally and spontaneously started by nurses themselves, to help their sisters when in difficulties.—Ed. *T. H.*]

### HOLIDAYS.

Miss E. Grace Jones, lady resident G.F.S. Home of Rest, Malvern Wells, writes: I see in The Hospital that two nurses are making inquiries about an economical holiday in September, and mountain scenery. I send you a report of our home. We have special terms—16s. per week for teachers and lady nurses. Our home is beautifully situated one and a-half miles from the small town of Malvern, and nearly a mile from the village of Malvern Wells. From our second storey we get on to the hill. I have had several nurses, and all have been most happy here. I can refer, of course, to ladies who have been here. With the exception of a cook and house-parlourmaid, we are voluntary workers, and my great aim is to make my home a real home of rest to tired workers.

[Intending visitors should write to Miss Jones, who will send them a printed form containing all particulars as to terms, &c.—Ed. *T.H.*]

### HYGIENE FOR NURSES.

A correspondent has pointed out an error of expression in the article on "Hygiene for Nurses" which appeared on May 30th. It is there properly stated that the intensity of radiant heat is in direct proportion to the temperature of its source, and inversely as the square of the distance from that source. But the example to illustrate this is wrongly worded, and should run as follows: A body, if removed to twice its distance from the source of heat will receive only one-fourth the heat, and if removed to four times the distance only one-sixteenth the heat which it received at first. Attention is also drawn to the fact that it is not exactly correct to say that "convection is peculiar to liquids." Convection can occur in all substances, the particles of which can move freely among one another—that is in all fluids. But the term fluid includes all gases—air for example—besides liquids. The warmth of a chimney-pot is as much due to the convection of heat by the fluid air as the warmth of a hot-water pipe is to convection by that other sort of fluid, "liquid" water.

### Where to Go.

Nurses' Co-operation, 8, New Cavendish Street.—An interesting "At Home" will be held by the Nurses' Co-operation on July 3rd at the Queen's Hall, Langham Place, from three to six p.m. The Meister Orchestra will perform, and a large and pleasant gathering may be anticipated.

Royal Hospital, Richmond.—The new "Princess May's Ward" for Children is to be formally opened on July 8th, at 3.45 p.m., by H.R.H. the Duchess of York. The Duke of York and other members of the Royal Family are also to be present. Ticket holders are urgently requested not to arrive later than 3.30 p.m.

British Home for Incurables, Streatham.—A garden party is to be held in the grounds of the Home, Crown Lane, Streatham, on Tuesday, June 30th, from 3 to 7 p.m. The Duchess of Sutherland has consented to open a sale of the inmates' work at 3.30 p.m. The Home will be open for inspection, and there will be music and entertainments during the afternoon. Tickets of invitation can be obtained from R. G. Salmond, Secretary, 72, Cheapside, E.C.

The Countess of Dufferin's Fund.—A garden fête and café chantant will be held at Kidbrook Lodge, St. German's Place, Blackheath, on Thursday, July 2nd, at 3 30. The opening ceremony will be performed by the Marchioness of Dufferin and Ava. The fête is under most distinguished patronage, and a large number of important artistes have promised their help. Should July 2nd prove inclement, the fête will take place on the following day. Tickets, price 3s., which admit to all entertainments, may be obtained from the hon. secretaries, Mrs. Hart, 2, Carisbrook Villas, Blackheath, S.E.; and Miss Edith Heather-Bigg, 14, Radnor Place, Hyde Park.

### Appointments.

Western Infirmary, Glasgow.—Miss Annie L. Strong has been appointed Matron of this hospital. Miss Strong has since 1893 held the appointment of Matron at the Royal Infirmary, Dundee.

Sunderland Nursing Institute.—Mrs. A. L. Marriner has been selected to fill the position of Lady Superintendent at this institute. She was trained at the Northern Hospital, Liverpool, and at Doncaster Infirmary, afterwards working as ward sister at St. Bartholomew's Hospital, London. Mrs. Marriner has also had considerable experience as Matron of the Clayton Hospital, Wakefield, and as Lady Superintendent at the New Sanatorium, Bradfield, Berks. We cordially wish her success in her new post.

Berwick Home and Hospital.—Miss Dora Lancaster has been appointed Lady Superintendent of this home. She received her training at the Meath Hospital and County Dublin Infirmary. Miss Lancaster has had a varied experience, having worked as a Red Cross sister at Dublin, as charge nurse at Bridgnorth and South Shropshire Infirmary, and as Queen's nurse at St. Patrick's Home, Dublin. She has also taken temporary duty as lady superintendent at St. Mark's Ophthalmic Hospital, Dublin.

### Minor Appointments.

Seamen's Hospital Society, Greenwich.—Miss Alice Mary Brown, Miss C. Graham, and Miss E. Wood-Woods have been appointed Sisters at the Seamen's Hospital, Greenwich. They received their training at the London Hospital, where Miss Brown was also for two years holiday and out-patient sister.

Lady Roberts' Nurses.—Miss A. J. Weighall has been appointed by Lady Roberts to the post of Sister in the officers' hospital, Murree, Punjab, India. Miss Weighall was trained at the Bristol Royal Infirmary, and has held the post of sister in the Military Hospital, Quetta, and at Sialkote. Miss Agnes Eddis has lately joined Lady Roberts' nursing staff. She was trained at St. Bartholomew's Hospital, and has excellent testimonials.

### The North-Eastern Hospital for Children.

### THE ROSE FETE.

The rose fête and bazaar held during three days of the present week at the Queen's Hall, Langham Place, has proved a great success. The arrangements reflect the greatest credit on the management, and the whole effect of the Hall, with its beautiful floral decorations, was most charming. The Duchess of Connaught performed the opening ceremony on the first day, the Duchess of Marlborough presided on the second, and the Duchess of Newcastle on the third day. At the conclusion of the ceremony on Wednesday the treasurer announced the gift of £100 by an anonymous donor, provided that another £100 was forthcoming before the close of the bazaar. It is expected that the nett proceeds will exceed £1,200, whilst about £800 was presented in purses to the Duchess of Connaught at the opening ceremoey.

### Notes and Queries.

### Queries.

(83) *Training.*—Will you kindly tell me where I may be trained as a nurse? I have excellent health, but fear I am over the usual age.—*M.*

(84) *Indian Nursing Service.*—Please tell me how to set about getting into the Indian Nursing Service?—*Sister May.*

(85) *Monthly Nursing.*—Can you tell me if any of the lying-in hospitals guarantee employment after training? How can I obtain admission into a general hospital as a probationer?—*Mrs. A.*

(86) *Midwifery Training.*—I wish to train as a midwife but have no idea how to set about making arrangements.—*E. M. D.*

(87) *Homes for Incurables.*—Will you give me a list of homes where a paralysed lady of limited means could be received? Country or seaside.—*Nurse.*

(88) *Address Wanted.*—Please tell me the address of the *Poor Law Officers' Journal* in Manchester?—*Nurse L. T.*

(89) *Private Nurses.*—Can you kindly tell me in what paper a letter appeared on this subject by Mr. Malcolm Morris, upon which comment was made in The Hospital some time in March or April.—*E. B.*

(90) *Carrying Chair.*—Please tell me the name of the maker of a lifting or carrying chair, mentioned in The Hospital a few weeks ago.—*Mrs. F. W., Hampstead.*

(91) *Private Nursing.*—Could you tell me the names of a few first-class homes in the West-end of London where a nurse taking her own fees could be received between her cases, and make it her permanent address?—*Black Poodle.*

(92) *Training.*—I should be glad to know if there are any hospitals in Liverpool where probationers are given a small salary during their training. I am very anxious to become a nurse, but cannot afford to give my services for the first year, as I find is some times required.—*Miss R.*

### Answers.

(83) *Training (M).*—You do not mention your age, so it is not easy to advise you. Ordinary, *i.e.*, paid, probationers are not admitted at most hospitals over 30, or at most 35 years of age. Paying probationers, who enter as a rule for a short period of training, are sometimes eligible over this age. You will find a complete list of hospitals, and the ages at which probationers are received, in "How to Become a Nurse" (Scientific Press, 428, Strand, W.C.).

(84) *Indian Nursing Service (Sister May).*—It would really be interesting to count the number of times this query is answered in this column during the course of a year. Write to the India Office, St. James's Park, S.W., for application form. Full particulars are given in Burdett's "Hospitals and Charities."

(85) *Monthly Nursing (Mrs. A.).*—Employment is certainly not guaranteed, but a register of monthly nurses and midwives is kept at most of the lying-in hospitals, upon which its pupils and nurses can enter their names. You should certainly obtain general training first, if you can. You will find a list of hospitals and training schools in Burdett's "Hospitals and Charities." Write to the matrons for particulars.

(86) *Midwifery Training (E. M. D.).*—Read the chapter on this subject in "How to Become a Nurse" (Scientific Press, 428, Strand, W.C.), and apply to the matrons of the various lying-in hospitals.

(87) *Homes for Incurables (Nurse).*—You will find a complete list of all such institutions in Burdett's "Hospitals and Charities."

(88) *Address Wanted (Nurse L. T.).*—71, Market Street, Manchester.

(89) *Private Nurses (E. B.).*—Mr. Malcolm Morris's article, "The Monstrous Regiment of Nurses," was published in the *Practitioner.* The date we do not know. Write to the publishers, Macmillan and Co., 29, Bedford Street, W.C.

(90) *Carrying Chair (Mrs. F. W., Hampstead).*—Write to Messrs. Fred. Fish and Sons, Suffolk House, Ipswich, for "Folding Cradle Stretcher."

(91) *Private Nursing (Black Poodle).*—The Nurses' Hostel, 27, Percy Street, Tottenham Court Road, is a hotel or boarding-house for nurses. You might also write to Miss Culverhouse, 18, Royal Avenue, Chelsea. There is a nurses' residential club, 92, Charlotte Street, Fitzroy Square; and there is a nurses' club at 17, Nottingham Place, W., manager, Mrs. Stubbs.

(92) *Training (Miss R.).*—Apply to the matrons at the Liverpool Royal Infirmary and the Liverpool Northern Hospital. At both these hospitals, we believe, probationers are paid from the beginning of their training. Excellent training is also to be had at the Mill Road Infirmary, Liverpool.

## Figures and Tables

**Fig. 24. f1:**
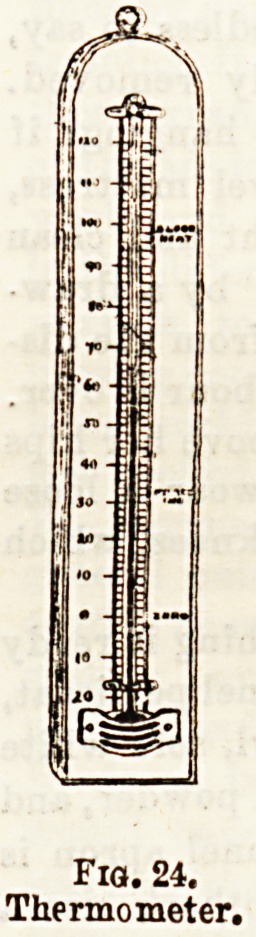


**Fig. 25. f2:**
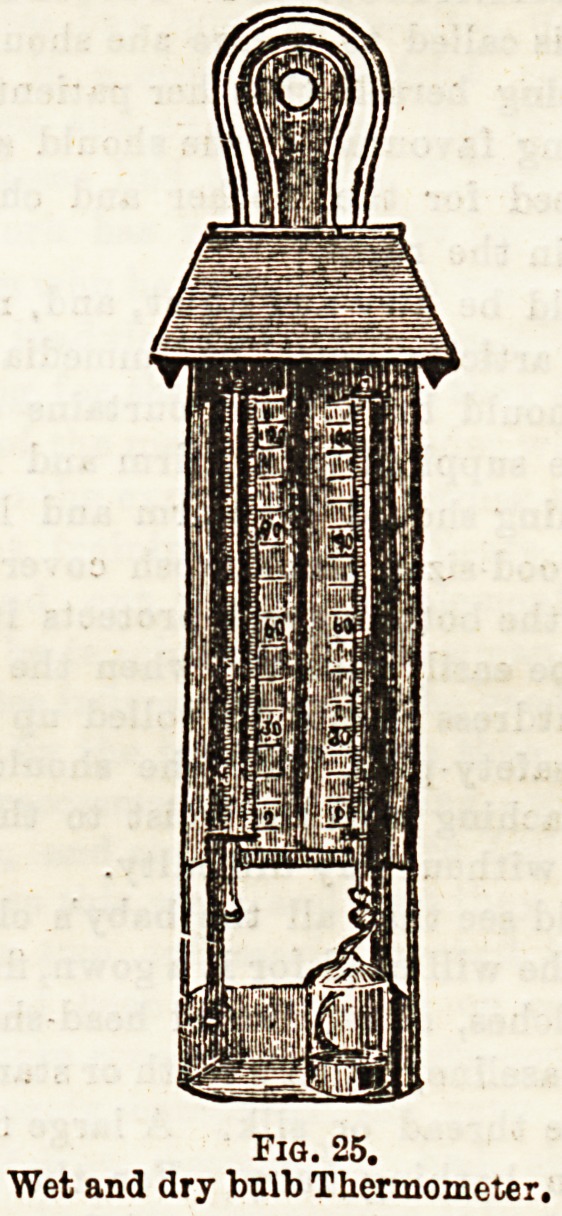


**Fig. 26. f3:**